# Polish Translation and Validation of the Mini Sarcopenia Risk Assessment (MSRA) Questionnaire to Assess Nutritional and Non-Nutritional Risk Factors of Sarcopenia in Older Adults

**DOI:** 10.3390/nu13041061

**Published:** 2021-03-24

**Authors:** Roma Krzymińska-Siemaszko, Ewa Deskur-Śmielecka, Arkadiusz Styszyński, Katarzyna Wieczorowska-Tobis

**Affiliations:** Department of Palliative Medicine, Poznan University of Medical Sciences, 61-245 Poznan, Poland; edeskur@ump.edu.pl (E.D.-Ś.); astyszyn@ump.edu.pl (A.S.); kwt@tobis.pl (K.W.-T.)

**Keywords:** sarcopenia, diagnosis, older adults

## Abstract

A simple, short, cheap, and reasonably sensitive and specific screening tool assessing both nutritional and non-nutritional risk factors for sarcopenia is needed. Potentially, such a tool may be the Mini Sarcopenia Risk Assessment (MSRA) Questionnaire, which is available in a seven-item (MSRA-7) and five-item (MSRA-5) version. The study’s aim was Polish translation and validation of both MSRA versions in 160 volunteers aged ≥60 years. MSRA was validated against the six sets of international diagnostic criteria for sarcopenia used as the reference standards. PL-MSRA-7 and PL-MSRA-5 both had high sensitivity (≥84.9%), regardless of the reference standard. The PL-MSRA-5 had better specificity (44.7–47.2%) than the PL-MSRA-7 (33.1–34.7%). Both questionnaires had similarly low positive predictive value (PL-MSRA-5: 17.9–29.5%; PL-MSRA-7: 14.4–25.2%). The negative predictive value was generally high for both questionnaires (PL-MSRA-7: 89.8–95.9%; PL-MSRA-5: 92.3–98.5%). PL-MSRA-5 had higher accuracy than the PL-MSRA-7 (50.0–55% vs. 39.4–45%, respectively). Based on the results, the Mini Sarcopenia Risk Assessment questionnaire was successfully adopted to the Polish language and validated in community-dwelling older adults from Poland. When compared with PL-MSRA-7, PL-MSRA-5 is a better tool for sarcopenia risk assessment.

## 1. Introduction

Sarcopenia, which means a muscle failure, is a severe condition threatening healthy aging, increasing the risk of falls and injuries, physical disability, dependence, and even death [1]. The diagnosis of sarcopenia is based on measurements of muscle mass and muscle strength and assessment of physical performance. The procedure is complex and time-consuming, and it requires highly specialized equipment and trained staff [2]. The high cost of professional devices precludes their widespread use in the assessment of muscle mass and strength in many countries, thus leading to underdiagnosis of sarcopenia [3]. Another reason can be the reluctance of health care professionals to perform another one laborious procedure in busy clinical practices [4]. The diagnosis of sarcopenia is further complicated by several existing definitions and diagnostic criteria and lack of a worldwide consensus [5]. Therefore, a simple, short, and cheap, yet reasonably sensitive and specific screening tool is needed in order to identify subjects with sarcopenia in a high-risk population [6].

To the best of our knowledge, two questionnaires have been developed for the screening of sarcopenia—the SARC-F questionnaire [7] and Mini Sarcopenia Risk Assessment (MSRA) [8]. The first one (SARC-F) was published in 2013 and it is widely used by researchers in the field. It contains five domains: (1) strength, (2) assistance with walking, (3) rising from a chair, (4) climbing stairs, and (5) falls. The SARC-F questionnaire has been translated into many languages, and the number of validation studies increases consecutively [9,10,11,12,13,14,15,16,17,18,19,20,21]. While the specificity of the SARC-F is very high, its sensitivity remains a concern, as documented by the meta-analysis that was published in 2018 [22]. The MSRA questionnaire’s sensitivity, published in 2017, is much higher than the SARC-F, yet its specificity is lower [23,24]. It includes seven simple questions assessing: (1) age, (2) the number of hospitalizations in the last year, (3) level of physical activity, (4) regular meal consumption, (5) the consumption of dairy products, (6) daily protein consumption, and (7) weight loss in the last year [8]. So far, the MSRA questionnaire has not been intensively used in research work: apart from the validation of the original version [8], we only found one study validating the Chinese language version [6].

Sarcopenia is a pluricausal condition. The useful clinical classification specifies two categories—primary and secondary sarcopenia [1,25]. Primary (otherwise age-related) sarcopenia appears with aging without any additional cause. It can be diagnosed when no apparent reason for sarcopenia can be identified in an older person. Secondary sarcopenia is diagnosed when a causal factor other than (or in addition to) aging can be found. These factors include chronic comorbidities (especially inflammatory diseases), low physical activity (sedentarity, immobilization due to disease or disability), nutrition-related issues (the insufficient intake of energy and/or protein), and poor nutritional status [26]. In the context of sarcopenia classification as a primary or secondary condition, the advantage of the MSRA questionnaire [8] over the SARC-F [7] consists in the assessment of sarcopenia risk factors other than physical fitness, such as regular meal consumption or adequate protein consumption necessary for muscle tissue maintenance. Additionally, the MSRA questionnaire also includes the adverse effect of body weight loss and frequent hospitalizations.

The aim of our study was an elaboration of the Polish language version of the MSRA questionnaire and validation against six sets of international diagnostic criteria for sarcopenia: the European Working Group on Sarcopenia in Older People (EWGSOP1) [25], the Extended European Working Group on Sarcopenia in Older People (EWGSOP2) [1], the Foundation for the National Institutes of Health (FNIH) Sarcopenia Project [27], the Asia Working Group for Sarcopenia (AWGS) [28], the International Working Group for Sarcopenia (IWGS) [29], and the Society on Sarcopenia, Cachexia, and Wasting Disorders (SCWD) [30]. To the best of our knowledge, it was the first validation study of the MSRA questionnaire using all of the currently available international algorithms for sarcopenia diagnosis in community-dwelling older adults. The original version of the MSRA questionnaire [8] was only validated against EWGSOP1 criteria [25], and the Chinese version of this tool was validated against four sets of criteria (EWGSOP1, AWGS, IWGS, and FNIH) [6].

## 2. Materials and Methods

### 2.1. Study Design and Participants

Translation and cross-cultural adaptation of the MSRA questionnaire was performed following the two phases World Health Organization (WHO) methodology for translating and intercultural adaptation of health questionnaires [31]. The study was conducted from July 2019 to February 2020. One hundred seventy community-dwelling volunteers that were living in Poznan, Poland were enrolled. The subjects were only included in the study if they were at least 60 years old, had normal cognitive function [defined as Abbreviated Mental Test Score [32] (AMTS) ≥ 7 points], and were able to walk a distance of 4 m (for gait speed measurement). As the body composition was assessed with the bioimpedance analyzer (BIA), subjects with cardiac pacemaker, metal implants, or oedemas were not included. Such contraindications were found in ten persons (two had a cardiac pacemaker, 5—AMTS score < 7, indicating a cognitive impairment, and three were not able to perform the 4-m usual gait speed test). 

Each subject gave written informed consent before entering the study, which was conducted under the Declaration of Helsinki. The Bioethics Committee of the Poznan University of Medical Sciences, Poland approved the study protocol (approval No: 1022/18).

### 2.2. Covariates and Data Collection

We performed a face-to-face interview with each participant to assess sociodemographic factors (age, sex, marital status, education level, and living conditions) and clinical data (self-reported comorbidity and the number of drugs taken regularly).

We used the Abbreviated Mental Test Score (AMTS) to assess subjects’ cognitive function [32]. 

The full version of the Mini Nutritional Assessment (MNA) questionnaire was used to evaluate subjects’ nutritional status [33]. Subjects’ functional fitness was assessed with the Katz scale for activities of daily living (ADL) [34] and the Lawton scale for instrumental activities of daily living (IADL) [35]. The detailed description of the tools that were used in our study can be found elsewhere [36].

In addition to the MSRA questionnaire, we estimated the risk of sarcopenia with the SARC-F questionnaire [7] and its modified version—the SARC-CalF [18]. We have described both of the questionnaires in our previous work [24].

### 2.3. Procedure for Translation and Adaption of the Mini Sarcopenia Risk Assessment (MSRA) Questionnaire

The validation process [31] was organized in two consecutive phases: (1) the translation of the questionnaire from English to Polish and cultural adaptation of this translation; and, (2) the clinical validation of the Polish MSRA to assess the performance of the MSRA questionnaire against the six sets of international diagnostic criteria of sarcopenia.

The MSRA questionnaire [8] has two versions (Table 1): the full form (including seven items, named MSRA-7) and the short form (including five items, named MSRA-5). The items of the MSRA-7 questionnaire are: (1) age, (2) the number of hospital treatment in the last year, (3) level of physical activity, (4) regular consumption of three meals a day, (5) consumption of dairy products, (6) consumption of protein, and (7) weight loss in the last year. Each item can be scored 0, 5, or 10, and the total score ≤ 30 indicates the risk of sarcopenia. Items (4) and (5) are not included in the MSRA-5 version. Each item of the MSRA-5 can be scored 0, 5, 10, or 15, and the total score ≤ 45 indicates sarcopenia risk.

A standardized forward-backward translation procedure was used to develop the Polish version of the MSRA [31]. A physician-geriatrician and a professional English translator, both Polish native speakers, independently translated the English version of the MSRA (original version) to Polish. The discrepancies between the two translations were discussed by a multidisciplinary expert panel, which consisted of an English translator, a physiotherapist, a dietician, and two physicians—specialists in geriatrics.

As a dish called ‘ragout’ is practically unknown in Poland, it was removed from the Polish version of the questionnaire. The word ‘ham’ was replaced with ‘cold meats’, as various types of sausages are popular in Poland. We also used the word ‘pulses’ instead of ‘legumes.’ In a satisfactory Polish version of MSRA-5 and MSRA-7, in the last question regarding weight loss in the last year, the second answer was expressed in the form: ‘no or ≤2 kg’ instead of ‘≤2 kg’.

An English native speaker fluent in Polish, who did not know the MSRA questionnaire, performed the back translation. The same expert panel compared the original and back-translated versions, and found no substantial differences.

We sent the back-translated questionnaires to Andrea Rossi, a co-author of the original MSRA, who accepted all changes and gave his consent to the Polish versions of the MSRA questionnaires. We obtained permission to use the prefix PL (for Poland) in the names of the evaluated questionnaires (PL-MSRA-7 and PL-MSRA-5), similarly to the prefix C in the Chinese versions (C-MSRA-7 and C-MSRA-5) [6]. Supplementary Table S1 presents Polish translated versions of PL-MSRA-7 and PL-MSRA-5.

The final Polish versions of MSRA-7 and MSRA-5 questionnaires were tested in 10 elderly subjects (five men and five women) with preserved mental function. The subjects were asked about perceived uncertainties concerning the questionnaire’s comprehension and cultural relevance. The inter-rater reliability and test-retest reliability of both MSRA versions was assessed by two independent researchers with a time interval of 2–4 weeks in 20 older subjects (10 men and 10 women). The number of respondents used in the inter-rater reliability and test-retest reliability assessment was based on the recommendations of the Special Interest Group on Sarcopenia of European Geriatric Medicine Society (EuGMS) for the validation studies of the SARC-F questionnaire [37], which we adapted for use in the current study.

### 2.4. Clinical Validation of the Translation of the Mini Sarcopenia Risk Assessment (MSRA) Questionnaire

#### 2.4.1. Assessment of Sarcopenia Using Six Sets of Different Diagnostic Criteria

A clinical validation study was performed to assess the performance of the Polish version of the MSRA-7 and MSRA-5. Owing to the lack of a worldwide consensus of diagnostic criteria for sarcopenia, we used six sets of international diagnostic criteria as reference standards: (1) the EWGSOP1 [25]; (2) the EWGSOP2 [1]; (3) the Foundation for the National Institutes of Health (FNIH) Sarcopenia Project [27]; (4) the Asia Working Group for Sarcopenia (AWGS) [28]; (5) the International Working Group for Sarcopenia (IWGS) [29]; and, (6) the Society on Sarcopenia, Cachexia and Wasting Disorders (SCWD) [30]. Table 2 provides the detailed description of measurements employed in each of the international sets of diagnostic criteria.

#### 2.4.2. Measurements of Muscle Mass, Muscle Strength, and Physical Performance

We used the BIA method to assess muscle mass (InBody 120, Biospace, Seoul, Korea) and two indices of low muscle mass, i.e. the Appendicular Lean Mass (ALM) index (ALM/height^2^) and the ALM/BMI index. 

We assessed the upper limb strength with a handgrip dynamometer (Saehan, Changwon, Korea) and the lower limb strength with the Chair Stand Test (CST). 

Physical performance was evaluated based on the 4-m usual gait speed test (UGS). The methodology of muscle mass, muscle strength, and physical performance has been described in detail in our previous publication [21]. Table 2 shows the cut-off points for all parameters.

#### 2.4.3. Assessment of Relationships between PL-MSRA and Other Measurements

The PL-MSRA-7 and PL-MSRA-5 questionnaires (each domain separately and total score) were validated against other functional and clinical measurements, such as age, HGS, CST, UGS, ALM, ALM/BMI, ALM index, BMI, MNA [33], ADL [34], and IADL [35]. We also used other sarcopenia diagnostic tools: the SARC-F [7] and SARC-CalF questionnaires [18], as an external reference for evaluating criterion-related validity.

### 2.5. Statistical Analysis

We used the STATISTICA 12.0 software (StatSoft, Cracow, Poland) to perform statistical analysis. The distribution of data was checked with the Shapiro–Wilk test. The analysis of continuous data (shown as mean ± standard deviation (SD)) was performed with the Student’s *t*-test, the Cochran–Cox test, or Mann–Whitney test. Categorical variables (shown as numbers (percentage)) were analyzed with the χ2 test with the Yates correction if applicable.

The first phase of the study consisted in the assessment of reliability of translation and cross-cultural validation of the MSRA. The MSRA total score was considered to be a numerical variable, and the intraclass correlation coefficient (ICC) was calculated for the inter-rater and test-retest reliability. The level of agreement was defined, as follows: poor <0.5, moderate 0.50–0.75, good 0.75–0.90, and excellent >0.90 [40].

The second phase of the study was aimed to assess the performance of the PL-MSRA-7 and PL-MSRA-5. To this end, five parameters were calculated: sensitivity (Se), specificity (Sp), positive predictive value (PPV), negative predictive value (NPV), and accuracy. The area under the curve (AUC) was assessed based on the receiver-operating characteristics (ROC) analysis. The diagnostic accuracy of the screening test based on AUC was defined, as follows: high >0.9, moderate 0.7 to 0.9, and low 0.5 to 0.7 [41].

Relationships between PL-MSRA-5 and PL-MSRA-7 (each domain separately and total score) and other functional and clinical measurements were investigated while using the Spearman correlation coefficients. We used the following cut-off points for the strength of correlation: very good if a correlation coefficient of 0.81–1.00, good if 0.61–0.80, moderate if 0.41–0.60, and poor to fair if <0.40 [42]. The difference between the frequencies of sarcopenia that were obtained by the PL-MSRA-7, PL-MSRA-5, and six sets of international diagnostic criteria of sarcopenia (EWGSOP1 [25], EWGSOP2 [1], FNIH [27], AWGS [28], IWGS [29], and SCWD [30]) were compared using the χ2. *p* < 0.05 was considered to be statistically significant.

## 3. Results

### 3.1. Characteristics of the Study Group

We enrolled 160 elderly persons (aged 60–93 years), of whom 56% were women. Table 3 shows the characteristics of the total study population and sex-based subgroups.

Men were younger than women (71.6 ± 7.6 vs. 73.5 ± 6.7 years, respectively; *p* = 0.08). Over 1/3 of the study population were subjects aged ≥75 years. Approximately 50% of the study population were married. Twice as many women were unmarried as men (*p* < 0.001). The vast majority of subjects had at least secondary education. Every third participant was living alone; women lived alone three times more frequently than men (*p* < 0.001).

Men were significantly higher (172.1 ± 6.4 vs. 156.8 ± 6.0 cm) and heavier (79.5 ± 14.0 vs. 66.8 ± 14.7 kg) than women, but the BMI was similar in both subgroups (26.8 ± 4.4 vs. 27.2 ± 6.1 kg/m^2^). The mean full MNA score in the total population indicated normal nutritional status. However, malnutrition or risk of malnutrition was diagnosed in 30% of the participants. No difference between men and women was found. Almost all of the participants were independent in activities of daily living assessed with the Katz scale. Men and women did not differ for the mean ADL and IADL scores. Based on face-to face interviews, the average number of chronic diseases was three, and regularly taken medicines was six. Participants most commonly declared hypertension (58.9%), chronic obstructive pulmonary disease (32.3%), cardiovascular disease (24.1%), dyslipidemia (20.3%), diabetes (18.4%), and osteoporosis/osteopenia (13.3%) (data available in 158/160 participants).

Women had significantly lower upper limb strength than men (19.2 ± 4.9 vs. 32.7 ± 9.1 kg, respectively; *p* < 0.001). In contrast, both of the subgroups had similar lower limb strength. The usual gait speed was somewhat higher in men (1.0 ± 0.3 vs. 0.9 ± 0.3 m/s, respectively; *p* = 0.09). Men had higher lean body mass and ALM index. Table 3 shows the mean scores obtained in questionnaires used in diagnostics for sarcopenia. While both sexes had similar scores in PL-MSRA-7 and PL-MSRA-5, women obtained higher scores in SARC-F (2.1 ± 2.0 vs. 1.4 ± 1.9 points, respectively; *p* < 0.05) and SARC-CalF questionnaires (4.6 ± 5.0 vs. 3.8 ± 5.4, respectively; *p* < 0.05).

Table 4 presents the results of the MSRA items. Almost two-thirds of the study group were aged 70 years or above. More than 1/3 of participants reported at least one hospitalization in the past year. Over 20% of the study group was unable to walk more than 1000 m. More than 15% of participants skipped a meal up to twice per week, and nearly 25% did not consume protein-rich products at least once a day (e.g., meat, eggs, pulses, milk, or dairy products). Over 1/3 of men and a similar proportion of women involved in the study lost at least 2 kg in the past year.

### 3.2. Prevalence of Sarcopenia

**Figure 1:**
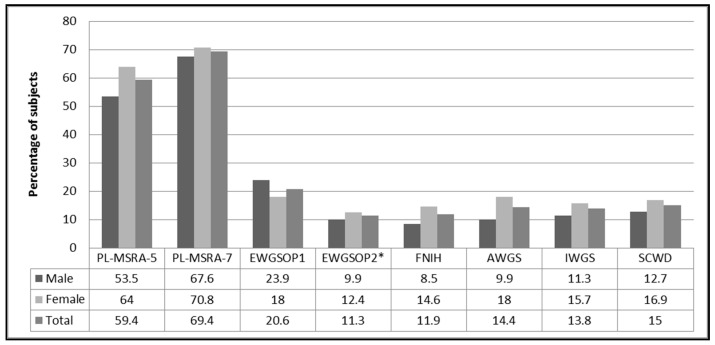
Prevalence rate (%) of sarcopenia according to the PL-MSRA and six sets of international diagnostic criteria for sarcopenia. Notes: * sarcopenia confirmed; Abbreviations: MSRA, Mini Sarcopenia Risk Assessment; EWGSOP1, the European Working Group on Sarcopenia in Older People 1; EWGSOP2, extended group for the European Working Group on Sarcopenia in Older People; FNIH, the Foundation for the National Institutes of Health; AWGS, Asian Working Group on Sarcopenia; IWGS, the International Working Group on Sarcopenia; SCWD, the Society on Sarcopenia, Cachexia and Wasting Disorders.

Figure 1 shows the frequency of sarcopenia in the total study group and both sexes. More than 2/3 of the study population were at risk of sarcopenia when the full version of the MSRA (PL-MSRA-7) questionnaire was used. The proportion was somewhat lower (approximately 60%) with the shortened version (PL-MSRA-5). The risk of sarcopenia was slightly higher in women (the difference was not significant). The prevalence of sarcopenia differed depending on the applied set of diagnostics criteria. It ranged from 11% when the EWGSOP2 [1] criteria were used, to 21% in the case of the EWGSOP1 criteria [25]. This difference was due, at least partially, to higher cut-off points for ALM index and handgrip strength in the EWGSOP1 criteria. With the exception of the EWGSOP1 criteria, the prevalence of sarcopenia was higher in women (the difference did not meet statistical significance).

### 3.3. Inter-Rater Reliability and Test-Retest Reliability

In order to assess inter-rater reliability, we calculated the ICC of the examinations performed independently by two researchers (a physician-geriatrician and a physiotherapist) in 10 men (mean age 74.6 ± 7.2 years) and 10 women (mean age 77.4 ± 7.5 years). We found an excellent agreement for the PL-MSRA-7 (0.910) and a good agreement for the PL-MSRA-5 (0.834).

One of the researchers (the physiotherapist) assessed the same 20 volunteers again at least two weeks (but no more than four weeks) later for the test-retest reliability check. For the PL-MSRA-7 questionnaire, the test-retest results differed in three cases; the test-retest agreement was 85%, and the test-retest intraclass correlation coefficient was 0.949 (*p* < 0.001), indicating excellent agreement. The test-retest scores of the PL-MSRA-5 version only differed in one subject, which resulted in excellent test-retest agreement (95%) and ICC (0.9799; *p* < 0.001).

### 3.4. Clinical Validation of the Polish MSRA Questionnaire Against a Different Reference Standard

The classification of sarcopenia using the PL-MSRA-7 and PL-MSRA-5 was tabulated according to the six different reference standards (Table 5). Table 6 shows the diagnostic values of the PL-MSRA-7 and PL-MSRA-5. The sensitivity ranged from 84.9% (EWGSOP1 criteria) to 91.7% (SCWD criteria) for the PL-MSRA-7 and from 84.9% (EWGSOP1) to 95.7% (AWGS) for the PL-MSRA-5. The specificity of both questionnaires was lower than its sensitivity. It ranged from 33.1% (EWGSOP2) to 34.7% (EWGSOP1) for the PL-MSRA-7, and from 44.7% (FNIH) to 47.2% (EWGSOP1) for PL-MSRA-5. Both of the questionnaires had similar PPV [PL-MSRA-5: 17.9% (FNIH and EWGSOP2)—29.5% (EWGSOP1); PL-MSRA-7: 14.4% (EWGSOP2)—25.2% (EWGSOP1)]. The negative predictive value was generally high for both questionnaires [PL-MSRA-7: 89.8% (EWGSOP1)—95.9% (for the remaining five sets of criteria); PL-MSRA-5: 92.3% (EWGSOP1)—98.5 (AWGS and EWGSOP2)]. PL-MSRA-5 had higher accuracy than the PL-MSRA-7 (50.0–55% vs. 39.4–45%, respectively). The AUC values for the PL-MSRA-7 ranged from 0.596 (FNIH criteria) to 0.685 (AWGS), which indicated low diagnostics accuracy. The PL-MSRA-5 questionnaire had moderate diagnostics accuracy (AUC ranging from 0.711 (EWGSOP1) to 0.759 (AWGS)).

### 3.5. Validity against Other Measurements

Table 7 shows the results of validation of MSRA questionnaires (each domain separately and total score) against other functional and clinical measurements (13 parameters). We found significant correlations between the PL-MSRA-7 and PL-MSRA-5 total score and other measurements, such as age, HGS, CST, USG, ALM, ALM index, BMI, SARC-F, SARC-CalF, IADL, and MNA. The Spearman correlations for PL-MSRA-7 ranged from −0.31 (for SARC-CalF) to 0.43 (for MNA) and from −0.377 (for SARC-CalF) to 0.526 (for MNA) for the PL-MSRA-5. There was a significant correlation between domain 3 of the MSRA (level of physical activity) and eight clinical and functional parameters (out of 13). A similar number of correlations was found for domain 7 (weight loss in the past year). No correlation was found between domain 5 (consumption of milk and dairy products) and the assessed parameters. Regarding criterion validity, the SARC-F and SARC-CalF scores significantly correlated with the PL-MSRA-7 and PL-MSRA-5. However, the strength of the correlation was poor to fair (<0.40).

## 4. Discussion

We cross-culturally adapted and validated the Polish version of the MSRA questionnaire, a sarcopenia screening tool elaborated in a seven-item (MSRA-7) and a five-item (MSRA-5) version. The design of our validation study was based on recommendations by Bahat et al. [37], formulated to guide studies on another sarcopenia risk assessment tool—the SARC-F questionnaire. Clinical validation studies should include elderly men and women able to walk independently and able to understand the aim and purpose of the study, according to these guidelines. We recruited 160 community-dwelling volunteers of both sexes aged 60–93. The prevalence of sarcopenia in our study population ranged from 11% when the EWGSOP2 [1] criteria were used to 21% in the case of the EWGSOP1 criteria [25]. These results are very similar to the values of 1–29% that were observed in the systematic review performed by Cruz-Jentoft et al. [43].

Using all of the currently available sets of international diagnostics criteria for sarcopenia, we found that both the full and shortened versions of the MSRA questionnaire have good sensitivity and are useful as a sarcopenia screening tool. Our findings are generally in accordance with previous studies [6,8]. PL-MSRA-7 and PL-MSRA-5 questionnaires both had high sensitivity (≥84.9%), regardless of the applied reference standard. Both of the versions had identical sensitivity against the EWGSOP1, FNIH, IWGS, and SCWD criteria, while PL-MSRA-5 had a higher sensitivity when EWGSOP2 and AWGS criteria were used. High sensitivity (80.4%) of the original MSRA-5 and MSRA-7 questionnaires was reported in a validation study conducted in 274 elderly inhabitants of Verona, Italy [8]. It should be noticed that the Italian analysis was performed against only one reference standard—EWGSOP1 criteria. Yang et al. performed another MSRA questionnaire validation study, using four sets of sarcopenia diagnostic criteria (EWGSOP1, AWGS, IWGS, and FNIH) [6] in 384 elderly inhabitants of Chengdu, China. The sensitivity of the C-MSRA-7 ranged from 78.0 to 86.9%, and that of the C-MSRA-5 from 80.2 to 90.2%. Unlike in our analysis, the C-MSRA-5 had higher sensitivity than the C-MSRA-7, except for FNIH criteria. In all of the validation studies (analyses of Rossi et al. [8], Yang et al. [6], and our study), the shortened version (MSRA-5) had better specificity and better overall diagnostics accuracy, regardless of the reference standard used. Therefore, MSRA-5 is a better screening tool for sarcopenia as compared to the MSRA-7. Moreover, it is more concise and less time-consuming, being a significant advantage in busy clinical practices.

As compared to the SARC-F questionnaire, the good point of the MSRA is the inclusion of nutritional items in screening for sarcopenia. Secondary sarcopenia may have multiple causes, with inadequate nutrition being among the most frequent. In our opinion, particularly important are MSRA questions concerning regular meals and intake of vegetable-based and animal-based proteins. It is well documented that both nutritional caloric intake and adequate amount, quality, and distribution of dietary proteins are important for the restraining of muscle mass loss [44,45,46,47]. The insufficient intake of protein and calories in the everyday diet increases the risk of malnutrition, not only in the elderly subjects, but also in younger age groups. Malnutrition in older people is usually associated with exacerbation of age-related muscle mass and strength loss. Thus, malnutrition increases the risk of sarcopenia and accelerates its progression. Vandewounde et al. described the relation between malnutrition and sarcopenia in 2012 [48], who suggested a new research problem—Malnutrition-Sarcopenia Syndrome. All of these findings indicate that sarcopenia may often be a nutrition-related disease [11,49].

Another important item in the MSRA questionnaire is weight loss in the past 12 months, and weight loss exceeding 2 kg is regarded as a risk factor for sarcopenia. Sarcopenia is particularly frequent in elderly subjects with unintentional weight loss [50]. However, an intentional weight loss (e.g., in obese elderly subjects), if not supervised by a dietician or physician, may reduce both fat and lean mass. The associated muscle mass loss may increase the risk of sarcopenia. Burgos Pelaez et al. [51] reported that elderly subjects have particular difficulties in regaining previously degraded muscles. While it is possible to control weight reduction therapy to maximally preserve muscle mass in older adults, it requires the careful balancing of energy supply (a small caloric deficiency) and protein intake (increase in high-quality protein and proper distribution), and the implementation of regular physical exercises [52]. The MSRA questionnaire does not specify if the loss of weight was intentional or unintentional. However, according to Coker and Wolfe [53], both weight loss types increase the risk of sarcopenia. We have not asked our study population about the reason for weight loss or verified the exact weight loss in the past year. Considering these topics in future research employing the MSRA questionnaire would help investigate the importance of body weight loss as a causal factor of sarcopenia. One-third of our study participants declared a weight decrease of at least 2 kg last year. In the study of Rossi et al. [8], the proportion of such subjects was even higher (52%). These findings demonstrate that weight loss is a common phenomenon in elderly subjects. It would be advisable, in our opinion, that a nutrition specialist should routinely control weight maintenance in such people.

The use of the BIA method for muscle mass measurement is an important limitation to our study. While it is well-known that most precise methods of muscle assessment are magnetic resonance imaging (MRI), computed tomography (CT), or dual energy X-ray absorptiometry (DEXA), these methods are difficult to use in larger populations due to their high cost and technical requirements [54]. DEXA is often used in clinical studies, but it should not be repeated more than twice per year due to X-ray irradiation. Moreover, its availability in sarcopenia diagnostics in our country is limited.

The assessment of muscle mass with non-invasive BIA method can be repeated without restrictions. The BIA method is simple and cheap, and the equipment is portable, which enables assessment in patient’s place of living.

A good point of our study is the use of all six currently available sets of international diagnostic criteria for sarcopenia as reference standards for MSRA validation: European Working Group on Sarcopenia in Older People 1 (EWGSOP1) [25], the Extended European Working Group on Sarcopenia in Older People (EWGSOP2) [1], the Foundation for the National Institutes of Health (FNIH) Sarcopenia Project [27], the Asia Working Group for Sarcopenia (AWGS) [28], the International Working Group for Sarcopenia (IWGS) [29], and the Society on Sarcopenia, Cachexia and Wasting Disorders (SCWD) [30].

## 5. Conclusions

The Mini Sarcopenia Risk Assessment questionnaire was successfully adopted in Polish and validated in community-dwelling older adults from Poland. Compared with PL-MSRA-7, PL-MSRA-5 is a better tool for sarcopenia risk assessment. As only three validation analyses (including the current one) have been performed so far, further studies are needed to demonstrate the MSRA is a useful screening tool for sarcopenia in clinical practice. Such analyses should preferably involve other settings (e.g., nursing home residents, hospitals) and comparisons between the MSRA and SARC-F questionnaires.

## Figures and Tables

**Table 1:** The Mini Sarcopenia Risk Assessment (MRSA) 7 and 5 items Questionnaire.

The MSRA-7 and MSRA-5 Questionnaires		
	MSRA-7 Score	MSRA-5 Score
1. How old are you?		
≥70 years	0	0
<70 years	5	5
2. Were you hospitalized in the last year?		
Yes, and more than 1 hospitalization	0	0
Yes 1 hospitalization,	5	10
No	10	15
3. What is your activity level?		
I’m able to walk <1000 m	0	0
I’m able to walk more than 1000 m	5	15
4. Do you eat 3 meals per day regularly?		
No, up to twice per week I skip a meal (e.g., I skip breakfast or I have only milk coffee or soup for dinner)	0	-
Yes	5	-
5. Do you consume any of the following?		
Milk or dairy products (e.g., yogurt, cheese), but not every day	0	-
Milk or dairy products (e.g., yogurt, cheese), at least once per day	5	-
6. Do you consume any of the following?		
Poultry, meat, fish, eggs, legumes, ragout, or ham but not every day	0	0
Poultry, meat, fish, eggs, legumes, ragout, or ham, at least once per day	5	15
7. Do you lose weight in the last year?		
>2 kg	0	0
no or ≤2 kg	5	10
sum of points		

Abbreviation: MSRA; Mini Sarcopenia Risk Assessment.

**Table 2:** Six sets of international diagnostic criteria for sarcopenia.

	Low Muscle Strength	Low Muscle Mass	Low Physical Performance	Diagnostic Criteria
Sarcopenia according to EWGSOP1	HGS < 30 kg for M HGS < 20 kg for W	ALM/height^2^ ≤ 7.40 kg/m^2^ for M * ALM/height^2^ ≤ 5.60 kg/m^2^ for W *	UGS ≤ 0.8 m/s for both sexes	HGS + LMM and/or UGS + LMM
Sarcopenia according to EWGSOP2	HGS < 27 kg for M HGS < 16 kg for W and/or CST > 15 s for five rises for both sexes	ALM/height^2^ ≤ 7.00 kg/m^2^ for M ALM/height^2^ ≤ 5.50 kg/m^2^ for W	─	HGS and/or CST + LMM
Sarcopenia according to AWGS	HGS < 26 kg for M HGS < 18 kg for W	ALM/height^2^ < 7.00 kg/m^2^ for M ALM/height^2^ < 5.40 kg/m^2^ for W	UGS ≤ 0.8 m/s for both sexes	HGS + LMM and/or UGS + LMM
Sarcopenia according to IWGS	─	ALM/height^2^ ≤ 7.23 kg/m^2^ for M ALM/height^2^ ≤ 5.67 kg/m^2^ for W	UGS < 1.0 m/s for both sexes	LMM + UGS
Sarcopenia according to FNIH	HGS < 26 kg for M HGS < 16 kg for W	ALM/BMI < 0.798 for M ALM/BMI < 0.512 for W	UGS ≤ 0.8 m/s for both sexes	HGS + LMM + UGS
Sarcopenia according to SCWD	─	ALM/height^2^ ≤ 7.29 kg/m^2^ for M ** ALM/height^2^ ≤ 5.52 kg/m^2^ for W **	UGS ≤ 1.0 m/s for both sexes	LMM + UGS

Notes: * Polish cut-off points for reference population aged 18–40 yrs [38]; ** Polish cut-off points for reference population aged 20–30 yrs [39]; Abbreviations: M, men; W, women; HGS, handgrip strength; ALM, appendicular lean mass; CST, Chair Stand Test; UGS, usual gait speed; BMI, body mass index; EWGSOP1, the European Working Group on Sarcopenia in Older People; EWGSOP2, extended group for the European Working Group on Sarcopenia in Older People; AWGS, Asian Working Group on Sarcopenia; IWGS, International Working Group on Sarcopenia; FNIH, Foundation for the National Institutes of Health; SCWD, Society on Sarcopenia Cachexia and Wasting Disorders.

**Table 3:** Characteristics of the whole study population and according to gender.

Characteristics	Total (*n* = 160)	Men (*n* = 71)	Women (*n* = 89)	*p*
Age (years) ^a^	72.6 (7.2)	71.6 (7.6)	73.5 (6.7)	0.0795
Age cohort				
65–74 yrs	101 (63.1)	48 (67.6)	53 (59.6)	0.2408
75 yrs or more	59 (36.9)	23 (32.4)	36 (40.4)	
Level of education ^b,&^				
no education or primary	7 (4.4)	1 (1.4)	6 (6.9)	0.2009
higher than primary	151 (95.6)	70 (98.6)	81 (93.1)	
Living conditions ^b,&^				
Living alone	50 (31.6)	11 (15.5)	39 (44.8)	0.0001
Living with others	108 (68.4)	60 (84.5)	48 (55.2)	
Marital status ^b,&^				
Unmarried	67 (42.4)	18 (25.4)	49 (56.3)	0.0001
Married	91 (57.6)	53 (74.6)	38 (43.7)	
Height (cm) ^a^	163.6 (9.8)	172.1 (6.4)	156.8 (6.0)	0.0000
Weight (kg) ^a^	72.4 (15.7)	79.5 (14.0)	66.8 (14.7)	0.0000
BMI (kg/m^2^) ^a^	27.0 (5.4)	26.8 (4.4)	27.2 (6.1)	0.6830
MNA score ^a^	24.9 (3.5)	25.1 (3.1)	24.7 (3.8)	0.8715
MNA status ^b^				
Malnutrition	6 (3.8)	1 (1.4)	5 (5.6)	0.3402
Risk of malnutrition	44 (27.5)	20 (28.2)	24 (27.0)	
Normal nutritional status	110 (68.8)	50 (70.4)	60 (67.4)	
Katz (ADL) score ^a^	5.8 (0.4)	5.8 (0.5)	5.7 (0.4)	0.0215
Katz (ADL), status ^b^				
Independent	158 (98.8)	70 (98.6)	88 (98.9)	0.5789
Partially dependent	2 (1.3)	1 (1.4)	1 (1.1)	
Dependent	0 (0.0)	0 (0.0)	0 (0.0)	
Lawton (IADL) score ^a^	25.5 (2.5)	25.3 (2.5)	25.7 (2.4)	0.2451
AMTS score ^a^	9.4 (0.6)	9.4 (0.7)	9.3 (0.6)	0.0578
Number of regular drugs ^a^	5.9 (3.9)	6.3 (4.0)	5.6 (3.9)	0.2651
Number of chronic diseases ^a,&^	3.3 (1.8)	2.8 (1.3)	3.7 (2.0)	0.0007
Handgrip strength ^a^	25.2 (9.7)	32.7 (9.1)	19.2 (4.9)	0.0000
Gait speed ^a^	1.0 (0.3)	1.0 (0.3)	0.9 (0.3)	0.0886
Chair stand test (s) ^a,^*	12.8 (4.6)	12.9 (4.8)	12.8 (4.5)	0.9769
ALM (kg) ^a^	19.3 (5.0)	23.5 (3.6)	15.8 (2.9)	0.0000
ALM index (kg/m^2^) ^a^	7.1 (1.2)	7.9 (1.0)	6.4 (1.0)	0.0000
Calf circumference ^a^	35.7 (3.8)	36.2 (3.4)	35.3 (4.0)	0.1043
PL-MSRA-7 score ^a^	28.4 (7.0)	28.6 (7.1)	28.3 (6.9)	0.6960
PL-MSRA-5 score ^a^	43.4 (11.9)	43.7 (12.6)	43.2 (11.4)	0.6211
SARC-F score ^a^	1.8 (2.0)	1.4 (1.9)	2.1 (2.0)	0.0107
SARC-CalF score ^a^	4.3 (5.2)	3.8 (5.4)	4.6 (5.0)	0.0333

Notes: ^a^ Data are presented as mean (standard deviation); ^b^ Data are presented as *n* (%); ^&^ data missing for two subjects; * *n* = 154, excluding six women who were unable to complete the CST due to low back pain; Abbreviations: BMI, body mass index; MNA, Mini Nutritional Assessment; ADL, activities of daily living; IADL, instrumental activities of daily living; AMTS, Abbreviated Mental Test Score; ALM, appendicular lean mass; MSRA, Mini Sarcopenia Risk Assessment.

**Table 4:** The answers to the Mini Sarcopenia Risk Assessment (MSRA) questions—the whole study population and according to gender.

MSRA-7 Items	Total (*n* = 160)	Men (*n* = 71)	Women (*n* = 89)	*p*
Q1.Age				
≥70 yrs	99 (61.9)	40 (56.3)	59 (66.3)	0.1978
<70 yrs	61 (38.1)	31 (43.7)	30 (33.7)	
Q2. Number of hospital treatment in the last year				
Yes, more than once	24 (15.0)	11 (15.5)	13 (14.6)	0.5764
Yes, once	37 (23.1)	19 (26.8)	18 (20.2)	
No	99 (61.9)	41 (57.7)	58 (65.2)	
Q3. Level of physical activity				
Able to walk less than 1000 m	36 (22.5)	18 (25.4)	18 (20.2)	0.4403
Able to walk more than 1000 m	124 (77.5)	53 (74.6)	71 (79.8)	
Q4. Regular consumption three meals a day				
No, up to twice a week, I skip a meal	25 (15.6)	8 (11.3)	17 (19.1)	0.1752
Yes	135 (84.4)	63 (88.7)	72 (80.9)	
Q5. Consumption of dairy products				
Yes, but not every day	38 (23.8)	20 (28.2)	18 (20.2)	0.2407
Yes, at least once a day	122 (76.3)	51 (71.8)	71 (79.8)	
Q6. Consumption of proteins				
Yes, but not every day	35 (21.9)	14 (19.7)	21 (23.6)	0.5556
Yes, at least once a day	125 (78.1)	57 (80.3)	68 (76.4)	
Q7. Weight loss in the last year				
>2 kg	54 (33.8)	21 (29.6)	33 (37.1)	0.3188
no or ≤2 kg	106 (66.3)	50 (70.4)	56 (62.9)		

Notes: Data are presented as *n* (%). Abbreviations: Q, question.

**Table 5:** PL-MSRA and various sarcopenia diagnostic criteria.

	PL-MSRA-5			PL-MSRA-7		
	Sarcopenia (*n* = 95)	No Sarcopenia (*n* = 65)	*p*	Sarcopenia (*n* = 111)	No Sarcopenia (*n* = 49)	*p*
EWGSOP1						
Sarcopenia	28 (29.5)	5 (7.7)	0.0008	28 (25.2)	5 (10.2)	0.0304
No sarcopenia	67 (70.5)	60 (92.3)		83 (74.8)	44 (89.8)	
EWGSOP2						
Sarcopenia	17 (17.9)	1 (1.5)	0.0013	16 (14.4)	2 (4.1)	0.0566
No sarcopenia	78 (82.1)	64 (98.5)		95 (85.6)	47 (95.9)	
FNIH						
Sarcopenia	17 (17.9)	2 (3.1)	0.0044	17 (15.3)	2 (4.1)	0.0429
No sarcopenia	78 (82.1)	63 (96.9)		94 (84.7)	47 (95.9)	
AWGS						
Sarcopenia	22 (23.2)	1 (1.5)	0.0001	21 (18.9)	2 (4.1)	0.0137
No sarcopenia	73 (76.8)	64 (98.5)		90 (81.1)	47 (95.9)	
IWGS						
Sarcopenia	20 (21.1)	2 (3.1)	0.0012	20 (18.0)	2 (4.1)	0.0183
No sarcopenia	75 (78.9)	63 (96.9)		91 (82.0)	47(95.9)	
SCWD						
Sarcopenia	22 (23.2)	2 (3.1)	0.0005	22 (19.8)	2 (4.1)	0.0102
No sarcopenia	73 (76.8)	63 (96.9)		89 (80.2)	47 (95.9)	

Notes: Data are presented as *n* (%); Abbreviations: MSRA, Mini Sarcopenia Risk Assessment; EWGSOP1, the European Working Group on Sarcopenia in Older People 1; EWGSOP2, extended group for the European Working Group on Sarcopenia in Older People 2; FNIH, the Foundation for the National Institutes of Health; AWGS, Asian Working Group on Sarcopenia; IWGS, the International Working Group on Sarcopenia; SCWD, the Society on Sarcopenia, Cachexia and Wasting Disorders.

**Table 6:** Sensitivity, specificity, positive and negative predictive values and receiver operating curve model of the PL-MSRA-5 and PL-MSRA-7 questionnaires against six sets of international diagnostic criteria of sarcopenia in the whole study population.

	Sensitivity (%)	Specificity (%)	PPV (%)	NPV (%)	Accuracy	AUC
**EWGSOP1**						
PL-MSRA-5	84.9 (68.1–94.9)	47.2 (38.3–56.3)	29.5 (25.1–34.2)	92.3 (84.0–96.5)	55.0 (47.0–62.9)	0.711 (0.614–0.807) *
PL-MSRA-7	84.9 (68.1–94.9)	34.7 (26.4–43.6)	25.2 (21.8–29.0)	89.8 (79.1–95.3)	45.0 (37.1–53.1)	0.649 (0.543–0.755)
**EWGSOP2**						
PL-MSRA-5	94.4 (72.7–99.9)	45.1 (36.7–53.6)	17.9 (15.3–20.8)	98.5 (90.4–99.8)	50.6 (42.6–58.6)	0.739 (0.643–0.836)*
PL-MSRA-7	88.9 (65.3–98.6)	33.1 (25.4–41.5)	14.4 (12.1–17.1)	95.9 (86.2–98.9)	39.4 (31.8–47.4)	0.655 (0.527–0.783)
**FNIH**						
PL-MSRA-5	89.5 (66.9–98.7)	44.7 (36.3–53.3)	17.9 (15.0–21.3)	96.9 (89.3–99.2)	50.0 (42.0–58.0)	0.717 (0.614–0.820)*
PL-MSRA-7	89.5 (66.9–98.7)	33.3 (25.6–41.8)	15.3 (13.0–18.0)	95.9 (86.1–98.9)	40.0 (32.4–48.0)	0.596 (0.486–0.707)
**AWGS**						
PL-MSRA-5	95.7 (78.1–99.9)	46.7 (38.2–55.4)	23.2 (20.1–26.5)	98.5 (90.3–99.8)	53.8 (45.7–61.7)	0.759 (0.674–0.845) *
PL-MSRA-7	91.3 (72.0–98.9)	34.3 (26.4–42.9)	18.9 (16.4–21.8)	95.9 (86.0–98.9)	42.5 (34.7–50.6)	0.685 (0.575–0.795)
**IWGS**						
PL-MSRA-5	90.9 (70.8–98.9)	45.7 (37.2–54.3)	21.1 (17.9–24.6)	96.9 (89.3–99.2)	51.9 (43.9–59.8)	0.747 (0.654–0.839) *
PL-MSRA-7	90.9 (70.8–98.9)	34.1 (26.2–42.6)	18.0 (15.5–20.8)	95.9 (86.0–98.9)	41.9 (34.1–49.9)	0.684 (0.574–0.795)
**SCWD**						
PL-MSRA-5	91.7 (73.0–99.0)	46.3 (37.7–55.1)	23.2 (19.8–26.9)	96.9 (89.2–99.2)	53.1 (45.1–61.1)	0.735 (0.645–0.824) *
PL-MSRA-7	91.7 (73.0–99.0)	34.6 (26.6–43.2)	19.8 (17.2–22.7)	95.9 (85.9–98.9)	43.1 (35.3–51.2)	0.667(0.560–0.774)

Notes: Data are presented with the 95% CI in parenthesis; * Significantly different (*p* < 0.05) with PL-MSRA-7; Abbreviations: PPV, positive predictive values; NPV, negative predictive values; AUC, area under the curve; EWGSOP1, the European Working Group on Sarcopenia in Older People 1; EWGSOP2, extended group for the European Working Group on Sarcopenia in Older People; FNIH, the Foundation for the National Institutes of Health; AWGS, Asian Working Group on Sarcopenia; IWGS, the International Working Group on Sarcopenia; SCWD, the Society on Sarcopenia, Cachexia and Wasting Disorders.

**Table 7:** Validation between the PL-MSRA-7 and PL-MSRA-5 (each domain and total score) and other related measurement.

	MSRA Q1		MSRA Q2		MSRA Q3		MSRA Q4		MSRA Q5		MSRA Q6		MSRA Q7		MSRA-7 Total Score		MSRA-5 Total Score	
	C	*p*	C	*p*	C	*p*	C	*p*	C	*p*	C	*p*	C	*p*	C	*p*	C	*p*
Age	−0.837	0.0000	0.077	0.3337	−0.101	0.2021	0.037	0.6412	0.119	0.1353	−0.163	0.0398	0.078	0.3247	−0.241	0.0021	−0.159	0.0451
HGS	0.214	0.0066	−0.055	0.4914	0.185	0.0194	0.019	0.8078	−0.048	0.5492	0.112	0.1575	0.076	0.3387	0.157	0.0479	0.176	0.0264
CST	−0.143	0.0765	−0.114	0.1579	−0.361	0.0000	0.124	0.1268	0.060	0.4607	0.148	0.0662	−0.161	0.0464	−0.157	0.0526	−0.264	0.0009
USG	0.146	0.0651	0.147	0.0635	0.376	0.0000	−0.071	0.3710	−0.036	0.6519	0.108	0.1739	0.184	0.0200	0.303	0.0001	0.331	0.0000
ALM/BMI	0.153	0.0527	−0.205	0.0093	0.038	0.6370	0.086	0.2822	−0.038	0.6376	0.131	0.0985	0.031	0.6980	0.049	0.5414	0.070	0.3781
ALM	0.129	0.1033	−0.072	0.3688	0.065	0.4167	0.110	0.1664	−0.125	0.1158	0.103	0.1959	0.222	0.0047	0.166	0.0361	0.206	0.0088
ALM index	0.104	0.1919	0.039	0.6265	0.074	0.3531	0.097	0.2238	−0.127	0.1085	0.088	0.2682	0.275	0.0004	0.211	0.0075	0.239	0.0023
BMI	−0.026	0.7455	0.179	0.0238	0.012	0.8756	0.024	0.7664	−0.059	0.4575	−0.018	0.8228	0.229	0.0035	0.158	0.0465	0.148	0.0621
SARC-F	−0.101	0.2038	−0.197	0.0127	−0.379	0.0000	0.014	0.8569	0.103	0.1965	0.061	0.4455	−0.027	0.7327	−0.208	0.0085	−0.297	0.0001
SARC-CalF	−0.078	0.3263	−0.242	0.0020	−0.390	0.0000	0.011	0.8885	0.112	0.1569	0.036	0.6490	−0.197	0.0126	−0.310	0.0001	−0.377	0.0000
ADL	0.237	0.0026	0.038	0.6339	0.211	0.0075	−0.090	0.2593	−0.092	0.2469	0.032	0.6845	−0.109	0.1683	0.063	0.4291	0.095	0.2308
IADL	0.160	0.0429	0.140	0.0776	0.415	0.0000	−0.116	0.1457	0.058	0.4659	0.024	0.7680	0.007	0.9267	0.251	0.0013	0.250	0.0014
MNA	−0.068	0.3936	0.327	0.0000	0.364	0.0000	0.236	0.0027	0.053	0.5028	−0.038	0.6374	0.296	0.0001	0.433	0.0000	0.526	0.0000

Abbreviations: Q, question; C, correlation; HGS, handgrip strenght; CST, Chair Stand Test; USG, usual gait speed; ALM, appendicular lean mass; BMI, body mass index; ADL, activities of daily living; IADL, instrumental activities of daily living; MNA, Mini Nutritional Assessment; MSRA, Mini Sarcopenia Risk Assessment.

## Data Availability

All relevant data are within the manuscript and are openly available in the Zenodo repository (DOI: 10.5281/zenodo.4483717).

## Supplementary Materials

The following are available online at https://www.mdpi.com/2072-6643/13/4/1061/s1, Table S1: Polish Version of Mini Sarcopenia Risk Assessment (MSRA) Questionnaire.
