# Supplementary material for: Polish Translation and Validation of the Mini Sarcopenia Risk Assessment (MSRA) Questionnaire to Assess Nutritional and Non-Nutritional Risk Factors of Sarcopenia in Older Adults
Source: Nutrients. 2021 Mar 24;13(4):1061. doi: 10.3390/nu13041061 (PMC8064359; doi:10.3390/nu13041061)
Supplement: Supplementary file 1 [file nutrients-13-01061-s001.pdf]

S1Table. Polish Version of Mini Sarcopenia Risk Assessment (MSRA) Questionnaire

**KWESTIONARIUSZ MSRA - KRÓTKA OCENA RYZYKA SARKOPENII**<br>**kwestionariusz 7- elementowy (PL-MSRA-7) i 5-elementowy (PL-MSRA-5)**		
**Elementy oceny**	**punktacja dla PL-MSRA-7**	**punktacja dla PL-MSRA-5**
**1. Ile Pan/Pani ma lat?**		
≥70 lat	0	0
<70 lat	5	5
**2. Czy był Pan/Pani leczony/a w szpitalu w ostatnim roku?**		
Tak, więcej niż jeden raz	0	0
Tak, jeden raz	5	10
Nie	10	15
**3. Jaki jest poziom Pani/Pana aktywności fizycznej?**		
Jest w stanie przejść mniej niż 1000 metrów	0	0
Jest w stanie przejść więcej niż 1000 metrów	5	15
**4. Czy regularnie zjada Pan/Pani 3 posiłki dziennie?**		
Nie, do dwóch razy na tydzień pomijam posiłek<br>(np. nie jem śniadania, ewentualnie piję tylko kawę/herbatę lub wypijam na obiad tylko kubek zupy)	0	-
Tak	5	-
**5.Czy spożywa Pan/Pani:**		
Mleko lub produkty mleczne (jogurt, ser), ale nie codziennie	0	-
Mleko lub produkty mleczne(jogurt, ser), co najmniej raz dziennie	5	-
**6.Czy spożywa Pan/Pani:**		
Drób, mięso/wędliny, ryby, jaja lub rośliny strączkowe, ale nie codziennie	0	0
Drób, mięso/wędliny, ryby, jaja lub rośliny strączkowe, co najmniej raz dziennie	5	15
**7.Czy schudł/a Pan/Pani w ostatnim roku?**		
>2 kg	0	0
nie lub ≤2 kg	5	10
**Suma punktów**		

Interpretacja wyniku:

- kwestionariusz MSRA ma dwie wersje: pełną (złożoną z 7 elementów, o nazwie MSRA-7) i krótką (złożoną z 5 elementów, o nazwie MSRA-5).
- wartości 0, 5 lub 10 punktów są przypisane do elementów w skali MSRA-7, podczas gdy wartości 0, 5, 10 lub 15 punktów są przypisane do elementów w skali MSRA-5.
- łączny wynik dla PL-MSRA-7 może wynosić minimalnie 0 punktów, a maksymalnie 40 punktów; wynik ≤30 punktów wskazuje na ryzyko sarkopenii
- łączny wynik dla PL- MSRA-5 może wynosić minimalnie 0 punktów, a maksymalnie 60 punktów; wynik≤ 45 punktów wskazuje na ryzyko sarkopenii
